# Early ART in Acute HIV-1 Infection: Impact on the B-Cell Compartment

**DOI:** 10.3389/fcimb.2020.00347

**Published:** 2020-07-16

**Authors:** Robert Badura, Russell B. Foxall, Dario Ligeiro, Miguel Rocha, Ana Godinho-Santos, Amelia C. Trombetta, Ana E. Sousa

**Affiliations:** ^1^Instituto de Medicina Molecular João Lobo Antunes, Faculdade de Medicina, Universidade de Lisboa, Lisbon, Portugal; ^2^Serviço de Doenças Infecciosas, Centro Hospitalar Universitário Lisboa Norte, Lisbon, Portugal; ^3^Centro de Sangue e Transplantação de Lisboa, Instituto Português de Sangue e Transplantação, IP, Lisbon, Portugal; ^4^Grupo de Ativistas em Tratamentos, Community Based Center for HIV and STD, CheckpointLX, Lisbon, Portugal

**Keywords:** B cells, HIV, severe acute HIV-1 infection, antiretroviral therapy, KRECs

## Abstract

HIV-1 infection induces B cell defects, not fully recovered upon antiretroviral therapy (ART). Acute infection and the early start of ART provide unique settings to address the impact of HIV on the B cell compartment. We took advantage of a cohort of 21 seroconverters, grouped according to the presence of severe manifestations likely mediated by antibodies or immune complexes, such as Guillain-Barré syndrome and autoimmune thrombocytopenic purpura, with a follow-up of 8 weeks upon effective ART. We combined B and T cell phenotyping with serum immunoglobulin level measurement and quantification of sj-KRECs and ΔB to estimate bone marrow output and peripheral proliferative history of B cells, respectively. We observed marked B cell disturbances, notably a significant expansion of cells expressing low levels of CD21, in parallel with markers of both impaired bone marrow output and increased peripheral B cell proliferation. This B cell dysregulation is likely to contribute to the severe immune-mediated conditions, as attested by the higher serum IgG and the reduced levels of sj-KRECs with increased ΔB in these individuals as compared to those patients with mild disease. Nevertheless, upon starting ART, the dynamic of B cell recovery was not distinct in the two groups, featuring both persistent alterations by week 8. Overall, we showed for the first time that acute HIV-1 infection is associated with decreased bone marrow B cell output assessed by sj-KRECs. Our study emphasizes the need to intervene in both bone marrow and peripheral responses to facilitate B cell recovery during acute HIV-1 infection.

## Introduction

HIV has become a controllable disease since the introduction of antiretroviral therapy (ART), due to the very efficient viral suppression and consequent recovery from the CD4 T lymphocyte depletion. Nonetheless non-AIDS related diseases persisted in long-term treated subjects, in association with increased levels of immune-activation and dysregulation. The initial viral dynamics, as much as the immediate HIV impact upon the immune system, is known to determine, by and large, the ensuing progression rate of the disease (Mellors et al., 1997). The early start of therapy in the acute HIV-1 infection is likely to reduce viral set point reservoirs and immune-activation (Jain et al., 2013). Therefore, there is an increasing interest in the study of these patients.

The fact that acute HIV infection (AHI) largely goes un-noticed has clearly hampered the investigation during this initial stage (Cohen et al., 2010), and justifies the relatively reduced number of studies. More than 50% of patients with acute HIV-1 infection have very few or no symptoms (Vanhems et al., 1998). Up to a third may present a more severe primary HIV-1 infection (Nicolás et al., 2019), such as opportunistic infections, neurological involvement, thrombocytopenic purpura and other serious conditions, with an increased risk of disease progression (Hoenigl et al., 2017). The pathophysiologic mechanisms leading to these manifestations are not fully clarified. Autoimmune diseases have been related to HIV-1 infection and the immune dysregulation observed in these patients (Virot et al., 2017). Among the postulated causes has been cited a direct viral pathologic effect of HIV linked to an aberrant immune activation which may lead to a serum sickness like syndrome (Lumsden and Bloomfield, 2016). Although the nadir of CD4 T cell count is predictive of the course of the disease after the initiation of ART (Pedersen et al., 1990; Hogg et al., 2001), it hardly describes the complete picture of immune dysregulation observed in acute HIV-1 infection (Ipp et al., 2014). Some acute phase severe clinical presentations with autoimmune-like characteristics are possibly mediated by antibodies or immune-complexes and might be related to B cell alterations.

Chronic HIV-1 infection is known to have an impact on the B cell compartment, resulting in non-specific polyclonal activation (Nagase et al., 2001), decreased B cell proliferative responses (Hart et al., 2007), loss of naïve and resting memory B cells (Carrillo et al., 2018), and higher percentages of atypical or exhausted B-cells (Moir et al., 2008), which are not fully recovered by ART (Richard et al., 2010). It has been shown that ART is less able to recover the depressed bone-marrow B cell output in chronic HIV-1 infections the later the treatment is started (Quiros-Roldan et al., 2012). The impact of a very early treatment approach in the acute HIV-1 infection upon bone-marrow output is so far unknown.

We have been gathering longitudinal data of seroconverters with severe clinical manifestations, like Guillain Barré syndrome, immune thrombocytopenic purpura, pericarditis, pneumonitis, rhabdomyolysis, along with data from mild or asymptomatic patients. This cohort represents a unique opportunity to investigate the B cell compartment in the early stages of the disease, and evaluate the impact that ART may have upon the putative B cell disturbances. We combined the study of the T and B cell subsets, with strategies to evaluate the B cell dynamics by the estimation of bone marrow output and peripheral proliferative history.

## Methods

### Patients and Study Design

**Figure 1:**
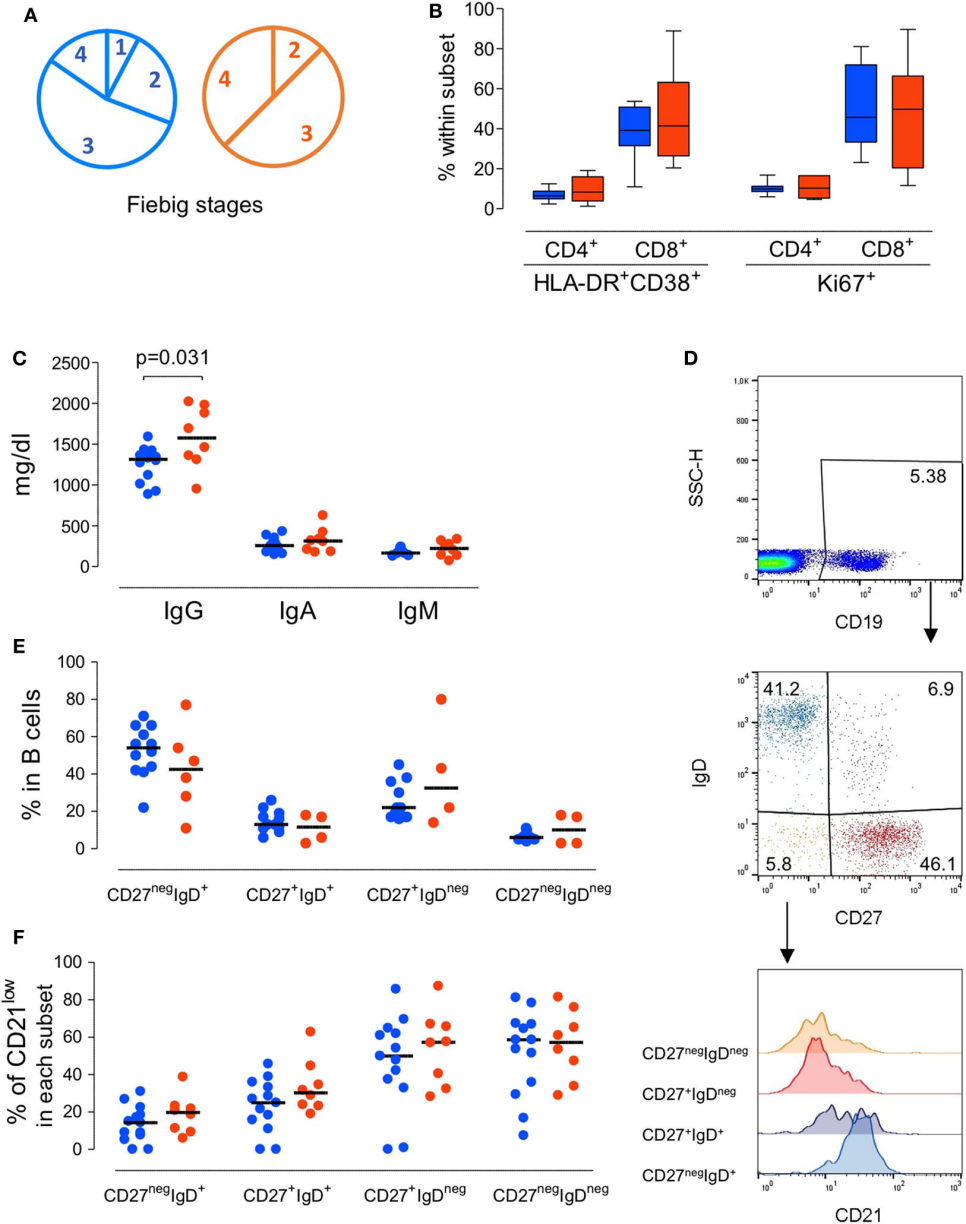
*Immunological profile of the patients at presentation grouped according to the severity of the clinical manifestations*. **(A)** Pie-charts of the patient distribution according to Fiebig scale. **(B)** Proportion of CD4 T cells and CD8 T cells expressing activation and/or cycling markers, namely concomitant expression of HLA-DR and CD38 or KI-67. **(C)** Serum Immunoglobulin levels. **(D)** Illustrative dot-plots of the B cell analysis by flow cytometry. **(E)** Frequency of subsets defined according to the expression of surface IgD and CD27 within total B cells. **(F)** Proportion of cells featuring low CD21 expression within each B cell subset. Each dot represents one individual and blue refers to subjects with mild symptoms and orange to those with severe manifestations. Bars represent median and *P* < 0.05 are shown.

An established network between the Department of Infectious Disease at the University Hospital of Santa Maria (Lisbon, Portugal) and the community based center for HIV testing in the same area (CheckpointLX) allowed the recruitment of 21 HIV-1 seroconverters with a very short interval between diagnosis and the suspected time of infection (33 days [26, 48]), who were studied prospectively from diagnosis up to 8 weeks after starting ART (Table 1). These patients were stratified at presentation, according to Fiebig stages (Fiebig et al., 2003), which subdivides acute infection into 5 stages according to the detection of HIV-RNA and the degree of Western blot's positivity, aiming to differentiate the successive phases of the acute infection. These stages are defined as follows: Fiebig 1: only viral RNA is detectable; Fiebig 2: both viral RNA and p24 antigen are detectable; Fiebig 3: one or two positive bands on a WB but not enough to declare positivity; Fiebig 4: all bands except p31 positive on a WB; Fiebig 5: is a fully positive WB. In agreement to this classification 11 of our patients had a Fiebig stage of 3 (see Table 1 and Figure 1). Patients were further grouped according to the clinical manifestations at presentation, namely: “Severe,” including 8 patients with organ specific or systemic disease requiring hospitalization; and “Mild,” the 13 subjects with the typical acute presentation being either asymptomatic or featuring self-limiting fever (for <5 days) with or without a mononucleosis like syndrome. All patients started ART, with the drug combinations listed in Table 1. Immunological and virological studies were performed immediately before starting ART (T0), and at 2 (T2), 4 (T4), and 8 (T8) weeks of treatment. Difference in sample numbers, in some time points was due to the necessity of having comparable timings among all patients, therefore some follow-ups were needed to be excluded if the patient did not appear for clinical evaluation and sample collection in the assumed week. This study was approved by the Ethical Board of the University Hospital of Santa Maria and the Faculty of Medicine of the University of Lisbon. All subjects provided written informed consent before data collection and blood sampling.

**Table 1:** Clinical and epidemiological data at baseline.

		**Age**	**Sex**	**Route**	**Symptoms**	**Fiebig**	**Days to ART**	**ART: TFV+FTC+**	**VL log10 cp/ml**	**Viral DNA cp/10^**6**^ cells**	**CD4 cells/μl**	**CD8 cells/μl**	**CD19 cells/μl**
Severe		29*	m	H	Severe hypoxic pneumonia	2	21	EFV	5.26	1,713	591	3,861	81
		65	m	H	Severe hypoxic pneumonitis	4	15	EFV	5.51	340	556	2,791	150
		31**	f	H	Rhabdomyolysis	3	18	EFV	7.04	43	220	623	125
		63	m	H	Guillain-Barre	4	48	DRV/r	4.94	1,310	919	2,280	54
		29	m	MSM	Pericarditis	4	17	EFV	6.55	NA	566	750	197
		57	m	H	Immune thrombocytopenic purpura	3	32	EFV	6.00	132	280	504	40
		29	m	MSM	Persistent fever and diarrhea	3	85	***+ RGV	6.26	NA	225	1,156	33
		39	m	H	Persistent fever	3	37	RGV	6.76	3176	425	343	190
	median	35	–	–	–	–	26.5	–	6.13	825	490	953	103
Mild		31	m	MSM	Fever and myalgia	4	20	EFV	5.49	778	417	1,737	67
		47	m	MSM	Mononucleosis-like syndrome	3	14	EFV	6.83	83,885	459	1,190	117
		32	m	MSM	Mononucleosis-like syndrome	4	32	RGV	4.37	12,163	448	916	54
		25	m	MSM	Adenopathies and asthenia	3	21	RGV	4.92	4,400	711	1,538	125
		40	m	MSM	Mononucleosis-like syndrome	3	18	RGV	5.45	321	329	529	60
		24	m	MSM	Fever and exanthema	3	31	RGV	5.41	1,260	619	2,292	104
		57	m	MSM	Fever and exanthema	2	14	RGV	7.00	2,097	397	561	88
		51	f	H	Fever and myalgia	2	14	RGV	7.00	106	513	344	32
		32	m	H	Fever and odynophagia	3	24	RGV	5.84	132	1,078	1,797	117
		23	f	H	No symptoms	1	/	RGV	6.39	113	474	374	58
		26	m	MSM	Adenopathies	3	32	RGV	6.01	9,454	835	1,185	74
		32	m	MSM	Fever and aphtosis	3	22	RGV	5.43	1,498	641	1,270	73
		35	m	H	Fever and exanthema	2	15	RGV	7.18	NA	470	607	110
	median	32	–	–	–	–	20.5	–	5.84	1,379	474	1,185	74

Additional therapies applied: ^*^Streptococcal pneumonia treated with ceftriaxone and ^**^Guillain-Barre syndrome treated with high dose Immunoglobulin. No statistical differences were found between the two groups. NA, not available; med, median; H, Heterosexual; MSM, Men having sex with men. Treatment was initiated in all patients as indicated in ART (antiretroviral therapy) with RGV, raltegravir or EFV, efavirenze or DRV/r, darunavir with ritonavir together with a backbone with TFV+FTC, tenofovir e emtricitabina, except as indicated in one patient ***were the backbone was constituted by ABV+3TC: abacavir and lamivudine; VL, viral load in Log10; DNA, for proviral DNA as copies of Gag/10^6^ cells.

### Cell Isolation and Flow Cytometry

Peripheral blood mononuclear cells (PBMC) were isolated from heparinized venous blood by Ficoll-Hypaque density gradient centrifugation (Gibco, Grand Island, New York, USA) washed and re-suspended at 1 × 10^6^ cells/ml in RPMI 1640 (Gibco). Samples were surface stained for 20 min at room temperature as previously described (Blanco et al., 2018, 2019), using the following anti-human monoclonal antibodies, with clone and fluorochrome specified in brackets: CD3 (OKT3, PerPC-Cy5.5); CD4 (SK3; PerCP), CD8 (SK1; allophycocyanin (APC-Cy7); CD19 (HIB19; PerCP-Cy5.5), CD38 (HB7; PE), CD45RA (HI100; APC), IgD (IA6-2; PE), HLA-DR (L243; FITC), CD27 (O323; APC), CD21 (BL13; FITC); Ki67 (B56; FITC), Samples were acquired on a 6-parameter FACSCalibur flow cytometer (BD Biosciences, San Jose, California, USA), with a minimum of 20,000 events analyzed for each parameter, and analyzed using FlowJo software (TreeStar, Inc., Ashland, Oregon, USA). Cells were successively gated on lymphocytes, according to forward/side scatter characteristics and B cells, as illustrated in Figure 1. T cell phenotype was assessed as previously described (Sousa et al., 2002). The absolute numbers of lymphocyte subsets were calculated by multiplying their frequency by the absolute lymphocyte counts obtained at the clinical laboratory at the same day of sampling.

### DNA Extraction and Total Viral DNA Quantification

DNA was extracted from PBMC (at least 0.5 × 10^6^), with DNAzol reagent (Life Technologies, Glasgow, UK). Digital PCR was performed using ddPCR Probe Supermix (Bio-Rad, California, USA) with 900 nM primers (2 pairs were used: set 1 – Primer Fw1: CGAGAGCGTCAGTATTAAGC; Primer Rv1: AGCTCCCTGCTTGCCCATAC; set 2 – Primer Fw2: CGAGAGCGTCGGTATTAAGC; Primer Rv2: AACAGGCCAGGATTAAGTGC), 250 nM probe (5′-FAM-CCCTGGCCTTAACCGAATT-MGB), and template DNA. Positive controls were generated from serial dilutions of plasmids containing the amplicons of HIV-1 gag and CD3 (a kind gift from Rémi Cheynier) (Fabre-Mersseman et al., 2011). Each 20 μL PCR reaction mixture was loaded into the Bio-Rad QX-200 emulsification device and droplets were formed following the manufacturer's instructions. The contents were transferred to a 96-well reaction plate and sealed with a pre-heated Eppendorf 96-well heat sealer for 5 s, as recommended by Bio-Rad. Total DNA was amplified separately in a T100TM Bio-Rad thermal cycler with the following cycling conditions: 10 min at 95°C, 45 cycles each consisting of a 30 s denaturation at 94°C followed by a 58°C extension for 60 s, and a final 10 min at 98°C. After cycling droplets were analyzed immediately.

### Quantification of sj-KRECs and Estimation of Peripheral B Cell Proliferation

A qPCR assay was conducted to determine the single joint (sj)-Kappa deleting recombination excision circles (KRECs) sequences in peripheral blood. The primers and probes used have been previously described (Serana et al., 2013). Briefly, 125 ng DNA was used for PCR amplification with 1x Taqman Universal Master Mix II (Applied Biosystems, Foster City, CA), 900 nM of 3'/5' outer primers and 250 nM of probes (FAM-TAMRA for TRAC and JOE-TAMRA for sj-KRECs). Sequence copy numbers were extrapolated from standard curves obtained by 10-fold serial dilutions of a plasmid, which contains KREC and TRAC fragments in a 1:1 ratio (a kind gift from L. Imberti, Spedali Civili of Brescia, Italy) (Serana et al., 2013). sj-KREC copies per μl of blood were calculated from the number of genome normalized sj-KREC molecules corrected for the number of white blood cells in peripheral blood (Chen et al., 2005). Finally, the number of B cell divisions was estimated as reported: somatic coding joints sequences cj-RssKde resulting from kappa-deleting rearrangement were quantified by qPCR, the difference of signal joint cycle threshold (Ct) to the coding joint Ct represents an estimate of divisions B cells had undergone (van Zelm et al., 2007, 2013). Threshold lines for Ct determination were positioned at the same level.

### Serum Immunoglobulin Levels

Total immunoglobulin G (IgG), immunoglobulin A (IgA) and immunoglobulin M (IgM) were quantified by immunonephelometry (Beckman-Coulter, Brea, California, USA) at the clinical laboratory of the Hospital de Santa Maria.

### Statistics

Statistical analyses were performed using SPSS version 24 and GraphPad/Prism version 7.0 (GraphPad Software, San Diego, California, USA). Mann-Withney U and Wilcoxon tests were used to compare unpaired and paired data, respectively. Anova and Mixed Anova tests were used for total and between group variance calculations in the follow-ups. Simple linear regression was performed to estimate associations of two numeric variables. Results are expressed as median, with interquartile range in [brackets], and *p* < 0.05 were considered to be significant.

## Results

We first evaluated whether patients with atypical/severe manifestations requiring hospitalization during acute HIV-1 infection present distinct immunological and/or virological features, as compared to those with the typical/mild presentation. As shown in Table 1, the severe group included 8 patients with clinical manifestations that are likely to be immune-mediated, namely Guillain-Barré syndrome, pneumonitis, pericarditis and autoimmune thrombocytopenic purpura. Notably, no opportunistic infections were reported. At enrolment the patients in the mild group (*n* = 13) were asymptomatic or had limiting symptoms, such as fever or a short mononucleosis-like syndrome. Distribution of Fiebig stages was dominated by stage 3 in both groups (Figure 1A), with no significant difference in distribution (*p* = 0.8), indicating that the differences in clinical manifestations were not related to the time since infection. Moreover, there were no differences in respect to age (*p* = 0.41), gender distribution (*p* = 0.85), or route of transmission between the two groups.

Strikingly, no significant differences were found in the levels of plasma viremia (5.84 and 6.13 Log, respectively, for mild and severe diseased patients groups, *p* = 0.86) or the cell associated total viral DNA (Table 1), suggesting that the degree of viral load was not the main determinant of the severity of the clinical manifestations.

Regarding the immune parameters, although there were three patients in the severe group with a profound CD4 depletion (<300 cells/μl), the described decline of CD4 T cell counts during the acute infection was observed in both groups (Table 1), with a respective median of 474 and 490/μl (*p* = 0.37). Moreover, the degree of the CD8 expansion was also comparable in the two groups (Table 1), leading to similar alteration of the CD4/CD8 ratio (0.60 [0.42–0.74] and 0.38 [0.19–0.70] for seroconverters with mild or severe symptoms, respectively, *p* = 0.16). In agreement, the markedly increased levels of CD4 and CD8 T cell activation were comparable between the two patient groups, despite their distinct clinical features (Figure 1B).

Concerning the B cell compartment, the “severe” group featured significantly higher levels of serum IgG (Figure 1C), mainly due to IgG_1_ (796 mg/dl [644–820] and 1,060 mg/dl [749–1,520] for mild and severe disease, respectively). This is particularly interesting because the documented clinical manifestations are thought to be largely immune-complex or antibody-mediated. This led us to analyse the B cell compartment in detail (Figure 1D). Both, the mild and severe group, featured reduced B cell counts. In fact, previously published data from the EuroFlow PID group, to which our lab contributed, reported in the 18 to 39 age group (the one most closely comparable to our patients age) a total number of 220 B cells/μl (median, range 41–470 and 54–438, 5th−95th percentile) (Blanco et al., 2018, 2019). These values, even taking in consideration the high variability of B cell count, are significantly different (*p* < 0.0001) compared to our patients' total B cell counts at baseline (median 80, range 32–197). Moreover, a similar distribution of the B cell subsets according to the expression of the memory marker CD27 and the surface levels of IgD was observed in the two groups of patients (Figure 1E), namely for IgD^+^CD27^neg^ or naive, IgD^neg^CD27^+^ or memory switched, IgD^+^CD27^+^ or memory unswitched, and IgD^neg^CD27^neg^ or other memory switched cells. Additionally, the percentage of cells expressing reduced levels of CD21 were expanded in all B cell subsets at baseline compared to the end of the follow up, irrespective of the clinical manifestations (Figure 1F). The increase in the CD21^low^ population percentage was particularly marked at baseline in switched B cells (median 50% in CD27^+^IgD^neg^CD21^low^ and 60% in CD27^neg^IgD^neg^CD21^low^) indicating high levels of B cell activation in both patient groups. Of note, the global analysis of the patients revealed a direct relationship between the frequency of CD21^low^ cells within switched memory (IgD^neg^CD27^+^) B cells and viremia (*r*^2^: 0.21/*p* = 0.04/slope: 0.11). Therefore, the B cell disturbances were not significantly different in the two groups of patients and appeared to be more closely related to viral load.

Importantly, upon starting ART there was a progressive improvement in all the clinical conditions paralleled by increased CD4 T cell count (*p* < 0.0001) and the effective control of viral load (with a median of 0 RNA copies/ml [0–65] and 18 RNA copies/ml [0-119] upon eight weeks of treatment for the mild and severe groups, respectively), *p* < 0.0001 (Supplementary Table 1).

**Figure 2:**
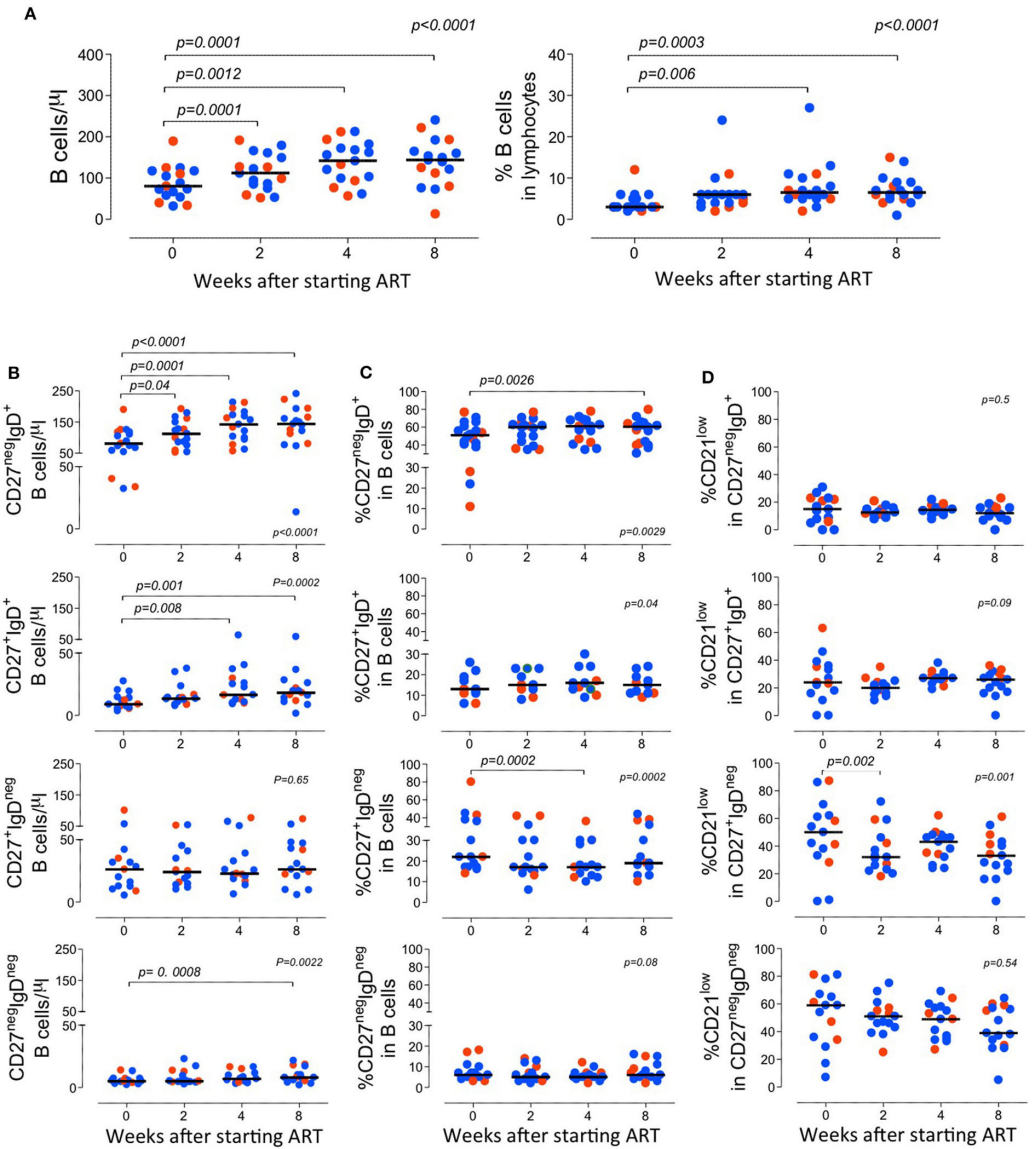
*Longitudinal analysis of the B cell compartment before and after starting ART*. **(A)** Total B cell counts (left graph) and frequency of B cells within lymphocytes (right graph). **(B–D)** B cell subsets were defined according to the expression of CD27 and IgD and graphs show the absolute counts of circulating subsets **(B)**; the proportion of each subset within total B cells **(C)**; and the frequency of B cells expressing low levels of CD21 within the subset **(D)**. Each dot represents one individual and blue refers to subjects with mild symptoms and orange to those with severe manifestations. Time 0 refers to evaluation immediately before starting ART and sub sequential analyses were performed at 2, 4, and 8 weeks upon therapy. Bars represent median. Anova for repeated measures and Dunn's Test were calculated. *P* < 0.05 are shown.

The variation of the B-cell frequency and absolute numbers where not different between the two groups (Figure 2). There was a progressive increase in the B cell counts, though they did not return to normal levels (Blanco et al., 2018, 2019), during the follow-up period (Figure 2).

**Figure 3:**
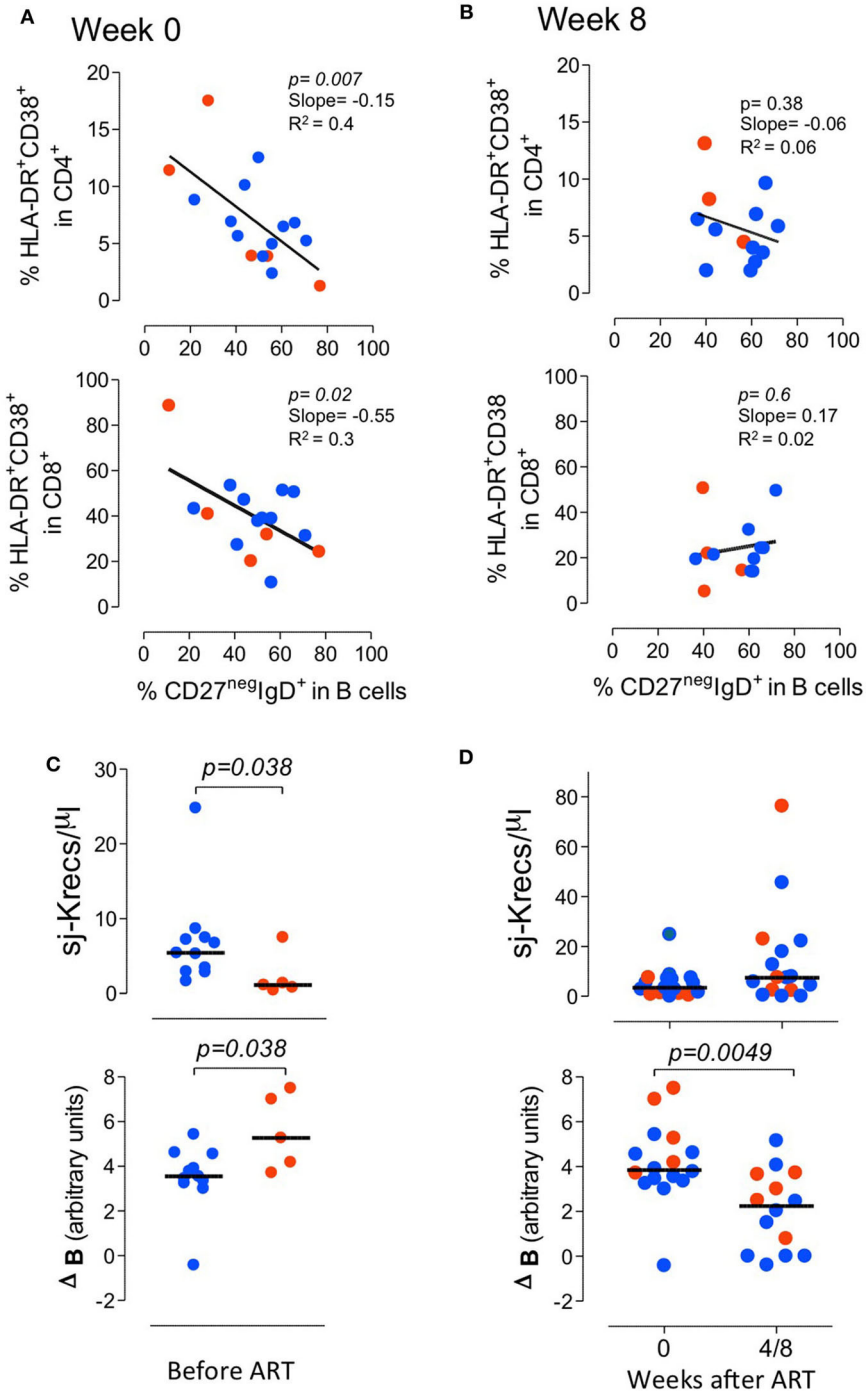
*Impact of immune activation and bone marrow output evaluated by KRECs in the B cell imbalances*. **(A)** Correlation between the frequency of HLA-DR+CD38+ cells within CD4 (top graph) and CD8 (bottom graph) T cells and the proportion of naive cells (CD27^+^IgD^neg^) within total B cells before treatment. **(B)** The same correlation after 8 weeks of ART. **(C)** Comparison of the levels of sj-KRECs and Delta B between patients with severe and mild disease before treatment. **(D)** Changes of sj-KREC and Delta B levels upon 4 or 8 weeks of ART. Each dot represents one individual and blue refers to subjects with mild symptoms and orange to those with severe manifestations. Bars represent median and *P* < 0.05 are shown.

It is known that immune activation is a key determinant of the B cell disturbances that characterize HIV infection (Moir and Fauci, 2014). In agreement, we observed a direct correlation between CD4 and CD8 T cell activation and the expansion of switched memory B cells (correlation between the frequency of CD27^+^IgD^neg^ within B cells and the proportion of HLA-DR^+^CD38^+^ cells within CD4 and CD8 T cells; *r*^2^: 0.2/*p*: 0.04/slope: 0.13, and *r*^2^: 0.45/*p*: 0.0016/slope: 0.7, respectively), as well as a negative correlation with frequency of naive B cells (correlation between the frequency of CD27^neg^IgD^+^ within B cells and the proportion of HLA-DR^+^CD38^+^ cells within CD4 and CD8 T cells; *r*^2^: 0.4/*p*: 0.007/slope: −0.15, and *r*^2^: 0.3/*p*: 0.02/slope: −0.55, respectively) (Figure 3A). Of note, no significant correlations were observed after 8 weeks of ART (Figure 3B), suggesting distinct dynamics of the recovery of the T and the B cell activation.

In order to further investigate the relative contributions of peripheral B cell proliferation induced by the immune stimulation and the possible impairment in B cell production in the bone marrow associated with the HIV infection, KRECs were quantified. Sj-KRECs are generated by the rearrangement of the Jκ-Cκ intron recombination signal sequence (intronRSS) to the kappa-deleting element which renders the IGK locus non-functional and precludes any further rearrangements in the IGK locus. Therefore, the coding joint of this rearrangement remains stably present in the genome. When a B lymphocyte with an intronRSS–Kde rearrangement divides, both daughter cells inherit the intronRSS–Kde coding joint in the genome. Conversely, the signal joint, which is on the episomal KREC, will be inherited by only one of the two daughter cells. As a consequence the difference between the kappa-deleting rearrangement and the corresponding excision circle (Delta B, ΔB) can be used as a measure for the *in vivo* replication history of an isolated B cell subset, assuming that the excision circle is a stable DNA structure, which is diluted 2-fold in every cell division. Since these rearrangements are terminated in the bone marrow, the quantification of sj-KRECs corresponds to B cell output from the bone marrow, and the calculated ΔB expresses their relative proliferation within the periphery. Before starting ART, the patients with severe manifestations featured significantly lower levels of sj-KRECs and higher levels of ΔB than those with mild disease (Figure 3C). These data suggest that severe disease was associated with both a more compromised bone marrow output and increased peripheral proliferation. Strikingly, upon ART there was a trend to a recovery of bone marrow output, as assessed by the sj-KRECS, as well as a significant decline in ΔB, supporting a reduction in peripheral proliferation in both groups of patients (Figure 3D).

Overall, the study of this unique cohort of acute HIV-1 infection suggests that severe disease is associated with an impairment in bone marrow output and more marked polyclonal B cell activation, as defined by higher IgG levels and higher ΔB. Notably, reduced B cell counts were a feature of acute HIV-1 infection irrespectively of disease severity. ART led to a recovery of total circulating B cells mainly due to an increase of the naïve B cell population. Moreover, 8 weeks of suppressive ART were unable to induce a full recovery of the memory switched B cell subset despite the contraction of the CD21^low^ population.

## Discussion

Our study confirms the importance of B cell disturbances in acute HIV-1 infection. The marked disruption of the B cell compartment was associated with impaired bone marrow output coupled with increased peripheral cell proliferation, as demonstrated via quantification of sj-KRECSs and ΔB for the first time in acute HIV-1 infected individuals. B cell dysregulation was likely contributed to the severe immune-mediated conditions observed in a subset of our patients, which featured lower sj-KRECs and increased ΔB and serum IgG levels, when compared to patients with mild disease.

B cell disturbances are well-recognized in untreated chronic HIV-1 infection (Cagigi et al., 2008; Amu et al., 2013). They are thought to be mainly linked to persistent immune activation (De Milito, 2004) and only partially recover upon ART initiation (Younas et al., 2016). The hallmarks of B cell perturbations are polyclonal hypergammaglobulinemia and expansion of B cells expressing low levels of CD21 (Fogli et al., 2012). Interestingly, these aberrant B cells are also increased in other conditions of chronic inflammation, such as autoimmune diseases (Thorarinsdottir et al., 2016; Das et al., 2018). Overall B-cells expressing low CD21 have a decreased proliferative capacity and effector function, as measured by decreased immunoglobulin diversification leading to the assumption that they do represent an exhausted B cell subpopulation (Moir et al., 2008). Moir et al. have further shown that they fail to respond to B cell stimuli in HIV-1- infected individuals (Moir et al., 2001). We showed that there is a linear association between viral load and memory switched B cells expressing low CD21 before therapy in acute HIV-1 infection.

Although part of the B cell responses are HIV specific, many indirect effects of the HIV infection that have been shown to contribute to the B cell stimulation (Schnittman et al., 1986; Kacani et al., 2000; Moir et al., 2000; Malaspina et al., 2006; Swingler et al., 2008; Haas et al., 2011; Ruffin et al., 2012; Sokoya et al., 2017). In fact, neither virema nor viral DNA level significantly differed between the two clinical groups, suggesting a contribution of other factors for the B cell dysregulation associated with severe manifestations. Curiously, HIV-2 infection, which is associated with low to undetectable viremia, and slow progressive CD4 decline in direct correlation with immune activation markers (Sousa et al., 2002), provides an example of profound B cell disturbances, namely marked depletion of both switched and unswitched memory B cells when compared to HIV-1 or seronegative controls (Tendeiro et al., 2012). This memory B cell reduction was mainly due to depletion of the class-switched subsets. CD27+IgD- depletion correlated with CD4+ T cell count in HIV-2 but not in HIV-1. A comparable loss of IgD+ memory B cell, correlating with markers of disease progression, like CD4 T-cell count and detectable viremia, was observed in both HIV-2 and HIV-1 chronically infected patients. Of note, we have also recently shown that follicular helper T cells (Tfh), the CD4 subset specialized in promoting B cell responses, supports productive HIV-2 infection and are an important reservoir in HIV-2-infected individuals (Godinho-Santos et al., 2020), suggesting that Tfh infection may contribute to the memory B cell imbalances.

An important finding of our study is the lack of full recovery of the switched memory B cell compartment upon 8 weeks of effective ART. Interestingly, Muir et al. suggested that HIV-1 induces disturbances in circulating Tfh function during the acute infection that may prevent the full recovery of the memory B cells upon treatment (Muir et al., 2016). Thus, preservation of Tfh function and B cell memory would require a very early start of ART, which would only be feasible with active prospective testing to identify seroconverters within the first week of infection (Ananworanich et al., 2016). Detailed functional studies of both the Tfh and the B cell compartments, ideally complemented with lymph node biopsies, are critically needed to understand the early HIV impact (Suresh et al., 2019).

The studies on the acute infection are always limited by the reduced number of subjects, though all the collected data are of high relevance. Given the rarity of acutely infected patients, we proritized the longitudinal analysis of the clinical and immunological data of seroconverters that consecutively presented to our clinic during a period of 90 months. Therefore, our study did not include the study of a group with chronic HIV infection in parallel and used the available data previously generated by us and others as a reference (Tendeiro et al., 2012; Blanco et al., 2018, 2019). Furthermore, differences in treatment regimens could have influenced the result. However, all patients showed decreased viremia and increased CD4^+^ counts at the end of 8 weeks observation, irrespective of which regimen they received. Given the relatively small number of patients, in this type of study it is difficult to investigate the influence of treatment regimens on the results. Even though all patients showed decreased viremia and increased CD4+ counts at the end of 8 weeks observation, irrespective of which regimen they received it is known that patients with full CD4 recovery and undetectable viremia may maintain some other immune disturbances after long-time ART. We believe that longer follow-up studies of acutely infected HIV patients therefore would be desirable. However, we report that several alterations related to B cell functions improved significantly, such as hyper-gammaglobulinemia and peripheral proliferation, as signified by the ΔB values. The profile of B cell recovery was not different in the two groups of patients, despite the association of severe disease with more pronounced B cell imbalances at presentation. Severe acute presentation has historically been linked to a more progressive disease (Hoenigl et al., 2017), nonetheless, larger follow up studies will be needed to demonstrate the impact of early treatment on improvement of prognosis.

Our study calls attention to the impact of HIV-1 acute infection in the bone marrow, although other factors, such as increased cell death, could have contributed to the observed levels of sjKRECs and ΔB, a question, which should be addressed in future studies. Bone marrow abnormalities are common in chronic HIV-1 infection and characterized by erythroid dysplasia and perturbations of myelopoiesis along with an increased plasma cell population (Tripathi et al., 2005; Alexaki and Wigdahl, 2008; Dhurve and Dhurve, 2013). The evaluation of sj-KRECs has previously revealed a decreased B cell output during chronic HIV-1 infection, that declines in direct association with the length of disease (Quiros-Roldan et al., 2012). Quantification of sj-KRECs represents, therefore, an important tool to address this aspect using peripheral blood (Verstegen et al., 2019).

Overall, we showed for the first time that acute HIV-1 infection is associated with decreased bone marrow B cell output assessed by sj-KRECs. This impairment is significantly more marked in patients with severe clinical manifestations, in parallel with increased peripheral B cell proliferation and higher levels of polyclonal IgG levels. Thus, bone marrow in addition to peripheral responses should be targeted to correct B cell dynamics in acute HIV-1 infection.

## Data Availability Statement

The datasets generated for this study are available on request to the corresponding author.

## Ethics Statement

The studies involving human participants were reviewed and approved by Ethical Board of the Centro Hospitalar Universitário Lisboa Norte (CHULN) and of the Faculty of Medicine of the University of Lisbon. The patients/participants provided their written informed consent to participate in this study.

## Author Contributions

RB, RF, and AS designed the study. RB, RF, and MR collected the clinical data. RF, DL, AG-S, and AT performed the immunological studies. AT, AG-S, RF, and RB analyzed data and investigated the selected variants. RB, AT, RF, DL, AG-S, and AS discussed the results. RB and AS supervised the study and wrote the paper. All authors contributed to the article and approved the submitted version.

## Conflict of Interest

The authors declare that the research was conducted in the absence of any commercial or financial relationships that could be construed as a potential conflict of interest.
