# Supplementary material for: Early ART in Acute HIV-1 Infection: Impact on the B-Cell Compartment
Source: Front Cell Infect Microbiol. 2020 Jul 16;10:347. doi: 10.3389/fcimb.2020.00347 (PMC7378391; doi:10.3389/fcimb.2020.00347)
Supplement: Supplementary file 1 [file Data_Sheet_1.PDF]

Supplementary Table 1: Statistical analysis of longitudinal data, for total patients and patient subgroups

	Time-point (weeks)				
**Viral load (copies/ml)**	**0**	**2**	**4**	**8**	**p-value**
**Total patients (n=21)**	1,001,000 (261,450-6,229,000)	NA	NA	0.0 (0-66)	<0.0001
**By group**					
**mild (n=13)**	694200 (261,450-8,349.000)	NA	NA	0.0 (0-60)	0.6
**severe (n=8)**	1,408,000 (216,332-5.209,000)	NA	NA	18 (0-119)	
**CD4 count (cells/ul)**	**0**	**2**	**4**	**8**	**p-value**
**Total patients (n=21)**	473.6 (220-1078)	718 (586-875)	690 (644-822)	773 (648-856)	0.0009
**By group**					
**mild (n=13)**	474 (433-676)	748 (623-895)	675 (634-835)	816 (686-871)	0.3
**severe (n=8)**	490 (239-585)	657 (449-848)	704 (633-828)	576 (440-678)	
**CD8 count (cells/ul)**	**0**	**2**	**4**	**8**	**p-value**
**Total (n=21)**	1156 (545-1767)	950 (653-1287)	833 (672-1221)	837 (672-11221	0.10
**By Group:**					
**mild (n=13)**	1185 (545-1638)	950 (683-1248)	853 (695-1193)	837 (679-1197)	0.29
**severe (n=8)**	953 (534-2663)	973 (617-1714)	749 (597-1089)	867 (496-1596)	
**CD4/CD8 ratio**	**0**	**2**	**4**	**8**	**p-value**
**Total (n=21)**	0.50 (0.31-0.73)	0.82 (0.54-1.06)	0.79 (0.62-1.14)	0.94 (0.59-1.23)	0.018
**By Group:**					
**mild (n=13)**	0.60 (0.43-0.74)	0.82 (0.57-1.04)	0.74 (0.62-1.03)	0.94 (0.68-1.23)	0.22
**severe (n=8)**	0.38 (0.19-0.71)	0.73 (0.31-1.22)	0.86 (0.69-1.27)	0.79 (0.31-1.28)	

Showing median values and corresponding interquartile range in brackets. The analysis for the total study-population states the significance difference of the evolution from 0 to 8 weeks (anova). Statistical analysis, referering to the groups (mixed anova), states the significance of the intergroup variation. NA: not applicable
